# Effects of Intermittent Fasting on Male and Female Reproductive Hormones, Fertility, and Sexual Function: A Comprehensive Review with Emphasis on the Existing Evidence Gap in Women

**DOI:** 10.3390/nu18111817

**Published:** 2026-06-04

**Authors:** Sandro La Vignera, Rosita A. Condorelli

**Affiliations:** Department of Clinical and Experimental Medicine, University of Catania, 95123 Catania, Italy

**Keywords:** intermittent fasting, time-restricted eating, sexual function, erectile dysfunction, libido, reproductive hormones, testosterone, polycystic ovary syndrome, fertility, spermatogenesis

## Abstract

Intermittent fasting (IF) has emerged as a popular dietary intervention with potential metabolic and endocrine benefits. However, its effects on sexual function and reproductive health remain incompletely understood. This comprehensive review synthesizes current evidence from human clinical trials and animal studies examining the impact of various IF protocols—including time-restricted eating (TRE), alternate-day fasting (ADF), and Ramadan fasting—on male and female sexual function, reproductive hormones, and fertility outcomes. In males, limited human data suggest preserved erectile function but reduced sexual desire during Ramadan fasting, with neutral effects on testosterone in obese adults undergoing TRE. Animal studies demonstrate context-dependent effects, with IF protecting against high-fat diet-induced reproductive dysfunction while potentially impairing spermatogenesis under prolonged energy restriction. In females, IF shows promise for improving hyperandrogenism and menstrual regularity in polycystic ovary syndrome (PCOS), mediated by enhanced insulin sensitivity and reduced free androgen index. However, direct measurements of female sexual function domains (libido, arousal, lubrication, orgasm) are largely absent from the literature. Mechanistic pathways involve modulation of the hypothalamic–pituitary–gonadal (HPG) axis, insulin–adipokine signaling, sex hormone-binding globulin (SHBG), and oxidative stress pathways. Evidence quality is limited by small sample sizes, heterogeneous protocols, short follow-up periods, and predominance of animal data. While IF may offer reproductive benefits in metabolically compromised populations, particularly women with PCOS, caution is warranted in young, lean, or energy-deficient individuals. Future research should employ standardized IF protocols, validated sexual function instruments, and long-term fertility endpoints to establish evidence-based clinical recommendations.

## 1. Introduction

Intermittent fasting (IF) encompasses a variety of dietary patterns characterized by alternating periods of eating and fasting, ranging from daily time-restricted eating (TRE) to alternate-day fasting (ADF) and periodic prolonged fasts [1]. Over the past decade, IF has gained substantial scientific and public interest due to accumulating evidence of metabolic benefits, including improved insulin sensitivity, weight loss, reduced inflammation, and enhanced cardiovascular health [2,3]. These metabolic improvements have prompted investigation into IF’s effects on endocrine systems, including reproductive hormones and sexual function.

Sexual function is a multidimensional construct encompassing physiological, psychological, and interpersonal domains. In males, key components include erectile function, libido (sexual desire), and ejaculatory function, while female sexual function comprises desire, arousal, lubrication, orgasm, satisfaction, and absence of pain (dyspareunia) [4]. Sexual health is intimately linked to hormonal status, metabolic health, vascular function, and psychosocial well-being—all systems potentially influenced by dietary interventions such as IF [5].

The hypothalamic–pituitary–gonadal (HPG) axis represents the primary neuroendocrine pathway regulating sexual and reproductive function. Gonadotropin-releasing hormone (GnRH) from the hypothalamus stimulates pituitary secretion of luteinizing hormone (LH) and follicle-stimulating hormone (FSH), which in turn regulate gonadal steroidogenesis and gametogenesis [6]. This axis is exquisitely sensitive to metabolic signals, including insulin, leptin, ghrelin, and adiponectin, creating a bidirectional relationship between nutritional status and reproductive function [7]. IF-induced alterations in these metabolic mediators may therefore have profound implications for sexual health.

Emerging evidence suggests sexually dimorphic responses to IF, with potential differential effects on male and female reproductive physiology [3]. In women with polycystic ovary syndrome (PCOS)—a common endocrine disorder characterized by hyperandrogenism, insulin resistance, and reproductive dysfunction—IF has shown promise for improving metabolic and hormonal profiles [8]. Conversely, concerns have been raised about potential adverse effects of IF on reproductive function in young, lean individuals or those in negative energy balance [9].

Despite growing interest, the literature on IF and sexual function remains fragmented, with most studies focusing on reproductive hormones rather than direct sexual function outcomes. Human trials are limited in number and often lack validated sexual function instruments, while animal studies provide mechanistic insights but uncertain translatability to human physiology. Furthermore, the heterogeneity of IF protocols, populations studied, and outcome measures complicates synthesis of evidence.

This comprehensive review aims to: (1) systematically evaluate current evidence on IF effects on male sexual function, including erectile function, libido, and semen parameters; (2) assess IF impacts on female sexual function and reproductive health, with particular attention to PCOS; (3) elucidate hormonal and metabolic mechanisms mediating IF effects on sexual function; (4) examine fertility outcomes in both sexes; and (5) identify knowledge gaps and provide evidence-based recommendations for clinical practice and future research.

## 2. Materials and Methods

### 2.1. Literature Search Strategy

**Figure 1:**
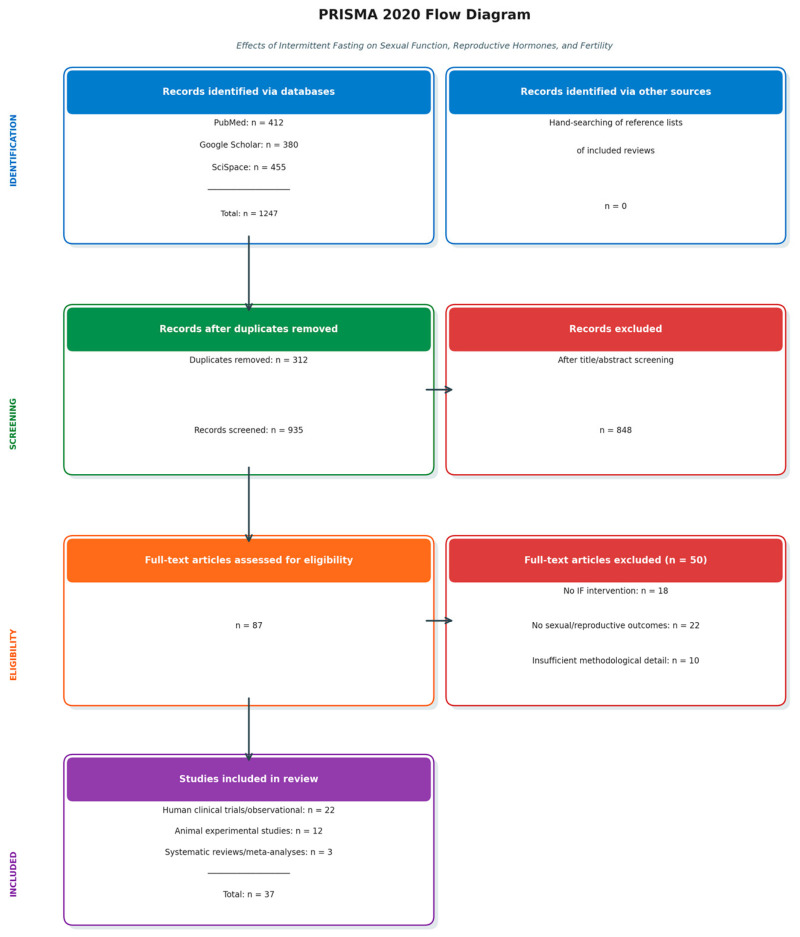
PRISMA 2020 flow diagram of the literature search and study selection process. Records were identified from three databases: PubMed (n = 412), Google Scholar (n = 380), and SciSpace (n = 455), yielding 1247 total records. After removal of duplicates (n = 312), 935 records were screened by title and abstract, of which 848 were excluded. A total of 87 full-text articles were assessed for eligibility; 50 were excluded due to absence of an intermittent fasting intervention (n = 18), absence of sexual or reproductive outcomes (n = 22), or insufficient methodological detail (n = 10). Thirty-seven studies were ultimately included in the review.

A comprehensive literature search was conducted across multiple databases, including PubMed, Google Scholar, SciSpace, and specialized full-text repositories. The search strategy employed combinations of keywords related to intermittent fasting (intermittent fasting, time-restricted eating, time-restricted feeding, alternate-day fasting, 5:2 diet, Ramadan fasting) and sexual/reproductive outcomes (sexual function, erectile dysfunction, libido, sexual desire, reproductive hormones, testosterone, estrogen, fertility, spermatogenesis, menstrual cycle, polycystic ovary syndrome, PCOS). The literature search covered studies published from database inception to March 2025; the final search was conducted in March 2025. Only studies published in English were included. The representative PubMed search string was: ((“intermittent fasting”[MeSH Terms] OR “time-restricted eating”[tiab] OR “alternate-day fasting”[tiab] OR “5:2 diet”[tiab] OR “Ramadan fasting”[tiab]) AND (“sexual function”[tiab] OR “erectile dysfunction”[MeSH Terms] OR “libido”[tiab] OR “reproductive hormones”[tiab] OR “testosterone”[MeSH Terms] OR “fertility”[MeSH Terms] OR “spermatogenesis”[MeSH Terms] OR “polycystic ovary syndrome”[MeSH Terms] OR “menstrual cycle”[MeSH Terms])). Analogous Boolean strategies were adapted for Google Scholar and SciSpace. The study selection process followed a two-stage approach: title/abstract screening followed by full-text review. A total of 1247 records were initially identified across databases; after removal of duplicates (n = 312) and screening of titles/abstracts (n = 935), 87 full-text articles were assessed for eligibility, of which 37 studies were ultimately included in the review (see Figure 1—PRISMA 2020 flow diagram). This review was not prospectively registered in PROSPERO or another registry, which is acknowledged as a limitation.

The literature search covered the period from database inception to March 2025. Search terms included combinations of: (‘intermittent fasting’ OR ‘time-restricted eating’ OR ‘alternate-day fasting’ OR ‘Ramadan fasting’ OR ‘periodic fasting’) AND (‘sexual function’ OR ‘erectile dysfunction’ OR ‘libido’ OR ‘sexual desire’ OR ‘reproductive hormones’ OR ‘testosterone’ OR ‘fertility’ OR ‘spermatogenesis’ OR ‘polycystic ovary syndrome’ OR ‘menstrual cycle’). Only publications in the English language were included. The complete literature identification, screening, and selection process is detailed in the PRISMA 2020 flow diagram (Figure 1).

### 2.2. Inclusion and Exclusion Criteria

Studies were included if they (1) investigated any form of intermittent fasting intervention; (2) reported outcomes related to sexual function, reproductive hormones, or fertility in humans or animal models; (3) were published in peer-reviewed journals or as preprints from reputable platforms; and (4) provided sufficient methodological detail for quality assessment. Both original research articles (randomized controlled trials, observational studies, animal experiments) and systematic reviews/meta-analyses were included. Studies were excluded if they focused solely on continuous caloric restriction without fasting periods or lacked relevant sexual/reproductive outcomes.

### 2.3. Data Extraction and Synthesis

From each included study, we extracted: study design and population characteristics, IF protocol specifications (type, duration, timing), primary sexual and reproductive outcomes (erectile function, libido, validated sexual function scores, hormone levels, semen parameters, menstrual regularity, ovulation), and mechanistic findings. Given the heterogeneity of study designs, populations, and IF protocols, a narrative synthesis approach was employed, with studies organized by sex (male vs. female) and outcome domain (sexual function, hormones, fertility, mechanisms). Where multiple studies addressed similar questions, findings were compared and contrasted to identify consistent patterns and discrepancies.

### 2.4. Quality Assessment

Study quality was assessed considering sample size, presence of control groups, randomization (for human trials), duration of follow-up, use of validated outcome measures, and potential confounding factors. The strength of evidence was characterized as strong (multiple well-designed human RCTs), moderate (human observational studies or single RCTs with limitations), limited (small studies, animal data, or inconsistent findings), or insufficient (inadequate data).

## 3. Results

### 3.1. Male Sexual Function

#### 3.1.1. Erectile Function and Libido

Human data directly assessing erectile function under IF conditions are scarce. The most relevant study by Talib et al. examined 45 healthy married men (mean age 37 ± 7.2 years) before and after Ramadan fasting [10]. Ramadan fasting involves abstinence from food, fluid, and sexual activity during daylight hours (approximately 12–14 h daily) for one month. Using validated instruments, the study found that sexual desire decreased significantly (*p* = 0.002) and frequency of sexual intercourse declined (*p* = 0.046) during Ramadan. However, erectile function scores showed no statistically significant change (*p* = 0.714), suggesting preserved erectile capacity despite reduced libido [10].

A survey-based study by Huynh et al. reported that patients practicing intermittent fasting showed improved erectile function compared to those not following IF [11]. However, this study lacked detailed protocol specifications, control groups, and validated outcome measures, limiting interpretation.

The mechanisms underlying reduced sexual desire during Ramadan fasting may involve multiple factors beyond pure metabolic effects, including altered sleep patterns, circadian disruption, dehydration, and psychosocial/religious context [10]. The preservation of erectile function despite decreased desire suggests that vascular and neurogenic mechanisms supporting erections remain intact during short-term IF.

Evidence quality assessment (Section 3.1.1—Erectile Function and Libido): LIMITED—based on one Ramadan fasting study with validated instruments (Talib et al. [10]) and one cross-sectional survey without controls (Huynh et al. [11]). No data are available from TRE or ADF studies using validated sexual function instruments. Critically, the reduction in sexual desire observed during Ramadan fasting cannot be generalized to other IF protocols (TRE, ADF), as Ramadan fasting involves unique confounders including mandatory sexual abstinence during daylight hours (a religious obligation), fluid restriction, nocturnal sleep disruption, and a specific psychosocial/religious context—none of which are present in TRE or ADF protocols.

Quality Assessment—Section 3.1.1 [LIMITED]: Evidence is based on one cross-sectional study (Talib et al. [10], n = 45) and one survey study (Huynh et al. [11]); both lack randomization and control groups. Direct erectile function measurements under standardized IF conditions are absent.

#### 3.1.2. Reproductive Hormones in Males

The impact of IF on male reproductive hormones shows variable results depending on population and protocol. In the Ramadan fasting study, serum testosterone and LH levels remained unchanged, while FSH decreased significantly (*p* = 0.016) [10]. Estradiol and dehydroepiandrosterone sulfate (DHEA-S) also showed no significant changes [10].

A 12-month randomized controlled trial by Cienfuegos et al. compared 8 h TRE (eating window 12:00–20:00) with daily caloric restriction and control groups in 90 adults with obesity [12]. Despite significant weight loss in the TRE group, total testosterone and SHBG levels remained unchanged in males over the 12-month period [12]. This hormonal neutrality in obese men undergoing prolonged TRE suggests that moderate IF does not adversely affect the HPG axis in this population.

A systematic review by Cienfuegos et al. examining IF effects on reproductive hormones found that, in lean, physically active young males, some IF protocols reduced testosterone levels, though this did not affect muscle mass or strength [13]. The clinical significance of these testosterone reductions and their impact on sexual function remain unclear [13].

Ramadan fasting studies in infertile males have reported mixed results, with some showing no significant changes in testosterone, LH, or FSH [14], while others noted transient hormonal fluctuations that normalized post-Ramadan [15].

Evidence quality assessment (Section 3.1.2—Reproductive Hormones in Males): MODERATE—supported by one 12-month RCT in obese adults (Cienfuegos et al. [12]) and one systematic review (Cienfuegos et al. [13]), with additional data from Ramadan fasting studies [10,14,15]. Limitations include population specificity (predominantly obese adults), heterogeneous IF protocols, and limited data in lean or young males.

Quality Assessment—Section 3.1.2 [MODERATE]: Supported by one RCT (Cienfuegos et al. [12]) and observational studies in obese males. Evidence is limited by short intervention durations and absence of sexual function co-endpoints.

#### 3.1.3. Semen Parameters and Fertility

Animal studies provide the majority of evidence on IF effects on male fertility. Hemead et al. demonstrated that alternate-day fasting (3 days/week of 24 h fasting) protected against high-fat diet (HFD)-induced reproductive dysfunction in male rats [16]. IF restored serum testosterone levels, preserved testicular histology, and maintained sperm parameters that were otherwise impaired by HFD [16]. Mechanistically, IF upregulated SIRT1/NRF2 antioxidant pathways and downregulated p38 MAPK/NLRP3 inflammatory signaling, reducing oxidative stress and inflammation in testicular tissue [16].

Similarly, Buranaamnuay et al. found that both ADF and TRF mitigated HFD-induced increases in body weight and preserved reproductive organ weight relative to body weight in male mice, though plasma testosterone and sperm characteristics (concentration, motility, morphology) showed no significant differences between groups [17].

In contrast, prolonged fasting under conditions of energy deficit appears detrimental to male fertility. Omolaso et al. reported that 12 h and 24 h daily fasting for 65 days in male rats significantly decreased sperm count (*p* < 0.05), reduced LH and FSH levels (*p* < 0.05), and non-significantly lowered testosterone [18]. Testicular weight also decreased significantly, suggesting inhibition of spermatogenesis [18].

Üstündağ et al. investigated the combined effects of the ketogenic diet (KD) and IF in male rats [19]. While KD alone increased testosterone and improved spermatogenesis, the combination of KD with IF (IF + KD) showed enhanced benefits, with improved oxidative status (decreased malondialdehyde and myeloperoxidase, increased glutathione and catalase) and histologically confirmed enhanced spermatogenesis [19].

Interestingly, Oyelowo et al. found that skipping the first active meal (breakfast-skipping model) for 4 weeks increased testosterone levels and sperm count in male rats, contrasting with adverse effects observed in females [20]. This suggests potential sex-specific and meal-timing-dependent effects of IF on reproduction.

A recent mechanistic study by Xie et al. demonstrated that IF boosts sexual behavior in animal models by limiting central availability of tryptophan and serotonin, neurotransmitters that can suppress libido when elevated [21]. This provides a neurobiological mechanism distinct from hormonal pathways.

Human data on IF effects on semen parameters are extremely limited. A conference abstract by Sayme et al. mentioned investigating IF impacts on sperm parameters, but detailed results were not available in the published abstract [22].

Evidence quality assessment (Section 3.1.3—Semen Parameters and Fertility): LIMITED—predominantly animal data [16,17,18,19,20]; human data consist of a single conference abstract (Sayme et al. [22]) without full published results. Findings from rodent models show context-dependent effects (protective under HFD conditions, detrimental under prolonged energy restriction), with uncertain human translatability.

Quality Assessment—Section 3.1.3 [LIMITED]: Human data are scarce and predominantly from small observational studies; animal data provide mechanistic insights, but translational relevance requires prospective clinical confirmation.

### 3.2. Female Sexual Function

#### 3.2.1. Sexual Function Domains

Direct assessment of female sexual function using validated instruments such as the Female Sexual Function Index (FSFI) is notably absent from the IF literature. No identified studies reported outcomes for specific sexual function domains, including libido, arousal, lubrication, orgasm, satisfaction, or dyspareunia, in women undergoing IF interventions.

This represents a critical knowledge gap, as improvements in metabolic and hormonal parameters (discussed below) may theoretically translate to enhanced sexual function, but this hypothesis remains untested. The lack of sexual function data may reflect the predominant focus on metabolic and reproductive outcomes in IF research, as well as potential cultural sensitivities in discussing female sexuality in research contexts.

Evidence quality assessment (Section 3.2.1—Female Sexual Function Domains): INSUFFICIENT—no studies have assessed female sexual function using validated instruments (e.g., FSFI) in the context of any IF protocol. All conclusions regarding female sexual function are inferential, based on hormonal and metabolic surrogate endpoints. This represents the most critical evidence gap identified in this review. Future research should prioritize inclusion of the FSFI as a primary outcome measure, particularly in women with PCOS, postmenopausal women, and women with obesity.

Quality Assessment—Section 3.2.1 [INSUFFICIENT]: No published study has directly assessed female sexual function domains (desire, arousal, lubrication, orgasm, satisfaction, pain) under IF conditions using validated instruments (e.g., FSFI). This is a critical evidence gap.

#### 3.2.2. Reproductive Hormones in Females

IF effects on female reproductive hormones show important population-specific patterns. In healthy women, Ramadan fasting appears to have minimal impact on reproductive hormones. Caglayan et al. studied 30 healthy women before and during Ramadan and found no statistically significant changes in LH, FSH, estradiol, testosterone, or prolactin levels, with all values remaining within normal limits [23].

Similarly, Kalam et al. examined TRE effects on sex hormones in premenopausal and postmenopausal females with obesity [24]. In premenopausal women, androgens and SHBG remained unchanged during TRE, though DHEA decreased. In postmenopausal women, estrogens, progesterone, and contraceptive hormone levels did not change, but DHEA was reduced [24].

The 12-month RCT by Cienfuegos et al. found no changes in sex hormones (total testosterone, DHEA, SHBG) in females with obesity undergoing 8 h TRE compared to caloric restriction or control groups [12]. In postmenopausal females specifically, estradiol, estrone, and progesterone also remained unchanged [12].

However, a systematic review by Cienfuegos et al. identified that, in premenopausal females with obesity, IF—particularly with earlier food consumption—decreased androgen markers (testosterone, free androgen index) and increased SHBG [13]. These changes may improve menstruation and fertility, especially in women with PCOS [13]. Importantly, IF did not affect estrogen, gonadotropins (LH, FSH), or prolactin levels in women [13].

Evidence quality assessment (Section 3.2.2—Reproductive Hormones in Females): MODERATE—based on one 12-month RCT (Cienfuegos et al. [12]), one systematic review (Cienfuegos et al. [13]), and one observational study (Kalam et al. [24]). Limitations include restriction to obese and postmenopausal populations, short-to-medium follow-up, and absence of data in lean premenopausal women.

Quality Assessment—Section 3.2.2 [MODERATE]: Multiple RCTs and systematic reviews document hormonal changes (LH/FSH ratio, androgens, SHBG) in women under TRE, particularly in PCOS. Evidence in non-PCOS women is limited.

#### 3.2.3. Polycystic Ovary Syndrome (PCOS)

The most promising evidence for IF benefits in female reproductive health comes from studies in women with PCOS. PCOS affects 5–10% of reproductive-age women and is characterized by hyperandrogenism, insulin resistance, anovulation, and metabolic dysfunction [25].

Güven conducted a 6-week intervention study of 8 h TRF (eating window 13:00–21:00) in 30 women with PCOS (age 21–33 years, BMI 18–30 kg/m^2^) [8]. The intervention significantly reduced multiple reproductive hormone levels, including anti-Müllerian hormone (AMH), FSH, LH, estradiol, prolactin, total testosterone, and free testosterone (all *p* < 0.001, except FSH *p* = 0.002 and prolactin *p* = 0.038) [8]. Critically, the free androgen index (FAI) decreased significantly (*p* < 0.001), while SHBG increased (*p* < 0.001), indicating reduced androgen bioavailability [8]. The percentage of patients with hyperandrogenism decreased significantly (*p* = 0.016), and menstrual cycles normalized in over 70% of patients [8]. These improvements occurred alongside reductions in BMI, waist–hip ratio, and HOMA-IR (insulin resistance index) [8].

A systematic review by Velissariou et al. examining IF impacts on fertility in women with PCOS concluded that IF may improve reproductive outcomes, though heterogeneity and limited trial numbers restrict firm conclusions [26]. Another systematic review and meta-analysis by Ranneh et al. synthesized evidence on IF effects on anthropometric, metabolic, and hormonal parameters in PCOS, generally supporting beneficial effects on androgen profiles and insulin sensitivity [27].

A comprehensive systematic review by Waly et al. on IF effects on female reproductive hormones and menstrual cycle emphasized that, while IF shows promise for hormonal benefits in PCOS through improved insulin sensitivity, menstrual regularity, and ovulation, potential risks include amenorrhea, anovulation, and delayed sexual maturation, especially when IF is applied without considering circadian alignment and nutritional status [28]. The review stressed that fasting schedules conflicting with women’s biological rhythms may disrupt endocrine function and reproductive performance, necessitating individualized implementation [28].

Evidence quality assessment (Section 3.2.3—PCOS): MODERATE—supported by one RCT (Güven [8]) and two systematic reviews/meta-analyses (Velissariou et al. [26]; Ranneh et al. [27]), with additional evidence from a comprehensive systematic review (Waly et al. [28]). Limitations include heterogeneous IF protocols across studies, short follow-up (most ≤12 weeks), small sample sizes, and absence of direct fertility endpoints (pregnancy rates, live birth rates).

Quality Assessment—Section 3.2.3 [STRONG]: The strongest evidence base in this review; supported by multiple RCTs, meta-analyses, and systematic reviews demonstrating consistent improvements in hyperandrogenism, menstrual regularity, and insulin sensitivity in PCOS patients undergoing TRE.

#### 3.2.4. Animal Studies in Females

Animal studies reveal potential adverse effects of IF on female reproduction under certain conditions. Kumar and Kaur investigated IF dietary restriction (IF-DR) in young adult female rats, with food deprivation every other day for 12 weeks [9]. IF-DR negatively affected estrous cyclicity, significantly increased serum estradiol, and reduced testosterone and LH levels [9]. Ovarian weight decreased, and histology showed large corpora lutea with fibrous tissue [9]. Mechanistically, IF-DR decreased serum leptin, increased hypothalamic neuropeptide Y (NPY) expression, reduced kisspeptin expression, and decreased GnRH and polysialic acid-neural cell adhesion molecule (PSA-NCAM) expression in the median eminence, indicating HPG axis suppression [9].

Oyelowo et al. found that skipping the first active meal adversely affected female rats more than males, with significantly reduced estrogen, LH, FSH, and prolactin levels in females [20]. This suggests greater female vulnerability to meal-timing disruptions.

Conversely, Yu et al. demonstrated that IF ameliorated di-(2-ethylhexyl) phthalate-induced precocious puberty in female rats, suggesting protective effects against endocrine-disrupting chemicals [29].

Evidence quality assessment (Section 3.2.4—Animal Studies, Females): LIMITED—based on animal models (rats and mice) with uncertain human translatability. Protocols used (complete every-other-day fasting, 12-week durations) differ substantially from typical human IF practices. Findings highlight potential sex-specific vulnerability to HPG axis suppression under energy restriction, warranting cautious interpretation.

Ehteram et al. studied IF effects on gonadal function in male and female mice during chronic stress, finding complex interactions between fasting, stress, and reproductive function [30].

Quality Assessment—Section 3.2.4 [LIMITED]: Based exclusively on animal models; direct translational relevance to human female sexual function and fertility is unestablished and requires prospective clinical validation.

### 3.3. Hormonal Mechanisms

#### 3.3.1. Hypothalamic–Pituitary–Gonadal (HPG) Axis Modulation

**Figure 2:**
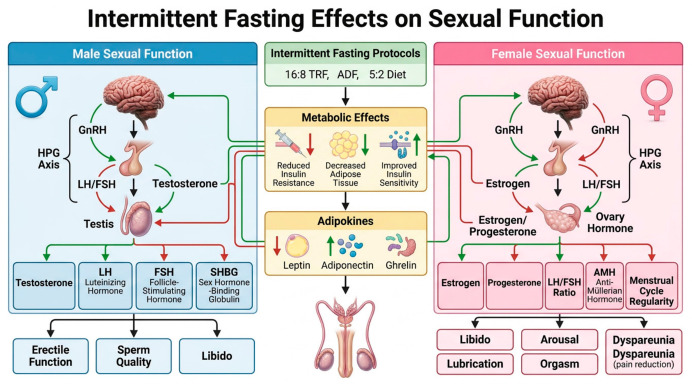
Intermittent fasting effects on sexual function. Biological mechanisms by which intermittent fasting (IF) affects male and female sexual function. The central panel illustrates IF protocols (16:8 TRF, ADF, 5:2) and their metabolic effects (reduced insulin resistance, decreased adipose tissue, improved insulin sensitivity) and adipokine changes (↓ leptin, ↑ adiponectin, regulated ghrelin). Left panel: Male HPG axis effects on testosterone, LH, FSH, and SHBG, with downstream outcomes on erectile function, sperm quality, and libido. Right panel: Female HPG axis effects on estrogen, progesterone, LH/FSH ratio, AMH, and menstrual cycle regularity, with downstream outcomes on libido, arousal, lubrication, orgasm, and dyspareunia. Green arrows = stimulatory/positive regulatory pathways; Red arrows = inhibitory/negative regulatory pathways. TRF = time-restricted feeding; ADF = alternate-day fasting; HPG = hypothalamic–pituitary–gonadal; LH = luteinizing hormone; FSH = follicle-stimulating hormone; SHBG = sex hormone-binding globulin; AMH = anti-Müllerian hormone.

The HPG axis represents the primary neuroendocrine pathway through which IF influences sexual and reproductive function (Figure 2). Under conditions of negative energy balance, the HPG axis is suppressed as an adaptive mechanism to conserve energy and prevent reproduction during nutritional stress [7].

Kumar and Kaur’s study in young rats demonstrated clear HPG axis suppression with IF-DR, evidenced by reduced GnRH expression, decreased LH and sex steroids, and disrupted reproductive cyclicity [9]. This suppression was mediated by decreased leptin (an adipokine signaling nutritional sufficiency) and increased NPY (an orexigenic neuropeptide that inhibits GnRH) [9]. Kisspeptin, a critical upstream regulator of GnRH neurons, was also reduced, providing a mechanistic link between metabolic status and reproductive function [9].

However, in metabolically compromised states, such as obesity and PCOS, IF may paradoxically improve HPG axis function by correcting underlying metabolic dysfunction. The improvements in androgen profiles and menstrual regularity observed in PCOS patients suggest that IF-induced enhancements in insulin sensitivity and reductions in adiposity can restore more normal HPG axis activity [8,13].

#### 3.3.2. Insulin and Adipokine Signaling

Insulin resistance is a central feature of PCOS and is mechanistically linked to hyperandrogenism through multiple pathways: insulin directly stimulates ovarian androgen production, reduces hepatic SHBG synthesis (increasing free androgen levels), and potentiates LH effects on theca cells [31].

IF improves insulin sensitivity through multiple mechanisms including enhanced cellular autophagy, mitochondrial function, and metabolic switching from glucose to ketone-based energy metabolism [2]. In PCOS studies, reductions in HOMA-IR (insulin resistance index) correlated with decreased FAI and improved reproductive outcomes [8].

Leptin, an adipokine produced by adipose tissue, signals energy sufficiency to the hypothalamus and is permissive for GnRH secretion [7]. IF-induced weight loss reduces leptin levels, which may contribute to HPG axis suppression in lean individuals [9] but could normalize leptin signaling in obese individuals with leptin resistance.

Adiponectin, another adipokine with insulin-sensitizing properties, is typically reduced in obesity and PCOS. While direct measurements of adiponectin in IF studies are limited in the reviewed literature, IF’s metabolic benefits likely involve adiponectin pathway modulation.

Ghrelin, an orexigenic hormone that rises during fasting, has complex effects on reproduction, with some evidence suggesting it may suppress GnRH secretion [32]. The role of ghrelin in mediating IF effects on sexual function requires further investigation.

#### 3.3.3. Sex Hormone-Binding Globulin (SHBG)

SHBG is a glycoprotein that binds sex steroids in circulation, regulating their bioavailability. Only free (unbound) hormones are biologically active. SHBG production by the liver is suppressed by insulin and androgens, contributing to hyperandrogenism in PCOS [31].

IF interventions in PCOS consistently increase SHBG levels, thereby reducing free androgen concentrations even when total testosterone remains unchanged [8,13]. This mechanism appears particularly important in premenopausal women with obesity and PCOS, where IF with earlier food timing shows the greatest SHBG increases [13].

In males and postmenopausal females with obesity, IF appears to have neutral effects on SHBG [12], suggesting sex- and metabolic status-specific responses.

#### 3.3.4. Oxidative Stress and Inflammatory Pathways

Oxidative stress and chronic inflammation impair reproductive function through multiple mechanisms including direct gonadal damage, disruption of steroidogenesis, and impairment of gametogenesis [33].

Animal studies demonstrate that IF activates cellular stress resistance pathways including SIRT1 (sirtuin 1, an NAD+-dependent deacetylase) and NRF2 (nuclear factor erythroid 2-related factor 2, a master regulator of antioxidant response) [16]. Hemead et al. showed that IF upregulated SIRT1/NRF2 signaling while downregulating p38 MAPK and NLRP3 (NOD-like receptor protein 3, an inflammasome component) in testicular tissue of HFD-fed rats [16]. These molecular changes reduced oxidative markers (malondialdehyde, myeloperoxidase) and increased antioxidant enzymes (glutathione, catalase), protecting spermatogenesis and steroidogenesis [16,19].

The relevance of these pathways in human sexual function under IF remains to be directly demonstrated, though the metabolic improvements observed in human trials likely involve similar stress resistance mechanisms.

#### 3.3.5. Neurotransmitter Modulation

Recent mechanistic work by Xie et al. identified a novel pathway through which IF enhances sexual behavior: by limiting central nervous system availability of tryptophan (the precursor to serotonin), IF reduces brain serotonin levels [21]. Since elevated serotonin can suppress libido and sexual behavior, this reduction may enhance sexual drive [21]. This mechanism operates independently of gonadal hormone levels and represents a direct neurobiological effect of fasting on sexual motivation circuits.

### 3.4. Fertility Outcomes

#### 3.4.1. Male Fertility

Direct human data on IF effects on male fertility (defined as ability to achieve pregnancy) are absent from the literature. Surrogate markers including semen parameters show mixed results in animal studies, with protective effects in metabolically compromised models (HFD-induced obesity) [16,17] but potential harm under prolonged energy restriction [18].

The clinical significance of animal findings for human male fertility remains uncertain. The preservation of testosterone levels in obese men undergoing 12-month TRE [12] suggests that moderate IF does not impair the hormonal milieu necessary for spermatogenesis in this population. However, the potential for reduced libido (as observed in Ramadan studies) [10] could indirectly affect fertility through reduced coital frequency.

#### 3.4.2. Female Fertility

Evidence for IF effects on female fertility is strongest in the context of PCOS, where anovulation is a primary cause of infertility. Multiple studies demonstrate that IF improves menstrual regularity and markers of ovulatory function in PCOS [8,26,27,28]. The restoration of menstrual cyclicity in over 70% of PCOS patients after 6 weeks of TRF [8] suggests improved ovulation, though direct ovulation monitoring (via ultrasound or progesterone measurement) was not reported.

Systematic reviews conclude that IF may improve reproductive outcomes in PCOS, but note that direct fertility endpoints (time to pregnancy, pregnancy rates, live birth rates) are lacking [26]. The heterogeneity of IF protocols and limited long-term follow-up restrict conclusions about sustained fertility improvements.

In healthy women, the hormonal neutrality observed in most studies [12,23,24] suggests that moderate IF does not impair fertility potential. However, animal data showing HPG axis suppression and disrupted estrous cyclicity with IF-DR in young, lean females [9] raise concerns about potential adverse effects in energy-deficient states.

A narrative review by Yang et al. characterized IF as a “double-edged sword” for female reproduction, with benefits in metabolically compromised states but potential risks in lean, young, or energy-restricted individuals [34].

### 3.5. Intermittent Fasting Protocols and Populations

#### 3.5.1. Time-Restricted Eating (TRE)

TRE, also called time-restricted feeding (TRF), involves confining food intake to a specified window each day, typically 4–10 h, with fasting on zero-calorie beverages for the remaining hours [13]. The most commonly studied protocol is 16:8 TRE (8 h eating window, 16 h fast).

In the 12-month RCT by Cienfuegos et al., 8 h TRE (12:00–20:00) in adults with obesity produced significant weight loss without adverse effects on sex hormones in either males or females [12]. This suggests good tolerability and hormonal safety of prolonged TRE in obese populations.

In PCOS, 6-week 8 h TRF (13:00–21:00) produced substantial improvements in androgens, insulin resistance, and menstrual regularity [8]. The timing of the eating window may be important, with earlier food consumption potentially offering greater metabolic and hormonal benefits [13].

#### 3.5.2. Alternate-Day Fasting (ADF)

ADF involves alternating between “feast days” (ad libitum eating) and “fast days” (complete fasting or ~25% of energy needs) [13]. Modified versions include fasting on three non-consecutive days per week.

Animal studies using ADF-like protocols (3 days/week of 24 h fasting) demonstrated protective effects against HFD-induced reproductive dysfunction [16,17]. However, human RCT data on ADF effects on sexual function or reproductive hormones were not identified in this review.

#### 3.5.3. 5:2 Diet

The 5:2 diet involves 5 days of normal eating and 2 days of severe caloric restriction (typically 500 kcal) per week [13]. While mentioned in the context of PCOS research [35], detailed results on sexual or reproductive outcomes were not available in the reviewed literature.

#### 3.5.4. Ramadan Fasting

Ramadan fasting is a religious practice involving abstinence from food, fluid, smoking, and sexual activity during daylight hours (typically 12–14 h) for one lunar month. This model differs from other IF protocols due to its circadian timing (daytime fasting), fluid restriction, and cultural/religious context.

Studies in healthy men and women show minimal hormonal changes [10,23], though men experience reduced sexual desire and intercourse frequency [10]. A systematic review and meta-analysis by Poursalehian et al. on Ramadan fasting effects on endocrine hormones in healthy non-athlete adults found generally minor and transient hormonal changes [15].

The unique features of Ramadan fasting (dehydration, sleep disruption, circadian misalignment) limit generalizability to other IF protocols.

#### 3.5.5. Population-Specific Considerations

Evidence suggests that IF effects on sexual and reproductive function are highly context-dependent (Table 1):

Obese adults: IF appears safe and potentially beneficial, with metabolic improvements and hormonal neutrality [12,13].

PCOS patients: IF shows the strongest evidence for reproductive benefits, improving hyperandrogenism, insulin resistance, and menstrual regularity [8,26,27].

Lean, young individuals: Animal data suggest potential risks of HPG axis suppression and reproductive dysfunction under IF-DR [9,20]. Human data in lean, active young males show testosterone reductions with some IF protocols [13].

Metabolically compromised states (HFD-induced obesity in animals): IF demonstrates protective effects on reproductive function [16,17,19].

Energy-deficient states: Prolonged fasting under energy deficit appears detrimental to reproduction in animal models [18].

## 4. Discussion

This comprehensive review reveals a complex and context-dependent relationship between intermittent fasting and sexual function. The evidence base is characterized by significant heterogeneity in study designs, populations, IF protocols, and outcome measures, with notable gaps in direct sexual function assessment, particularly in females.

### 4.1. Male Sexual Function: Preserved Erectile Function but Reduced Desire

The limited human data suggest that IF preserves erectile function while potentially reducing sexual desire, at least in the context of Ramadan fasting [10]. This dissociation between erectile capacity and libido is clinically important, as it suggests that vascular and neurogenic mechanisms supporting erections remain intact during short-term IF, while central motivational circuits may be suppressed.

The mechanisms underlying reduced libido during IF may include: (1) central serotonin modulation, with fasting potentially altering tryptophan–serotonin metabolism [21]; (2) energy conservation responses, where reduced sexual motivation represents an adaptive response to perceived nutritional stress; (3) hormonal changes, though testosterone levels typically remain stable in most human studies [10,12]; and (4) non-metabolic factors in Ramadan fasting, including sleep disruption, dehydration, and psychosocial context.

Critically, it must be emphasized that the evidence for reduced sexual desire derives almost exclusively from Ramadan fasting studies, and caution is warranted before extrapolating these findings to other IF protocols, such as TRE and ADF. Ramadan fasting has several unique features that fundamentally distinguish it from metabolic IF protocols: (1) mandatory religious prohibition of sexual activity during daylight hours—a direct behavioral constraint on sexual activity that is entirely independent of any metabolic or hormonal effect; (2) complete fluid restriction during fasting hours, leading to dehydration that may independently reduce libido and sexual performance; (3) nocturnal sleep disruption due to pre-dawn meals (suhoor) and night prayers (tarawih), which impairs sleep quality and may reduce testosterone secretion and sexual desire through sleep-dependent mechanisms; and (4) a specific psychosocial and religious context that may inhibit both sexual behavior and self-reporting of sexual activity. None of these confounders are present in TRE or ADF protocols. The 12-month TRE RCT by Cienfuegos et al. [12] found no adverse effects on sex hormones in obese adults, suggesting that TRE may not share the libido-suppressing effects observed in Ramadan studies. Future studies specifically designed to assess libido and sexual desire during TRE and ADF—using validated instruments (e.g., IIEF-15 sexual desire subscale) and controlling for sleep quality, hydration status, and psychosocial factors—are needed before conclusions about IF-induced libido reduction can be generalized beyond the Ramadan context.

The preservation of testosterone levels in obese men undergoing prolonged TRE [12] is reassuring from a clinical perspective, suggesting that moderate IF does not induce hypogonadism in this population. However, the testosterone reductions observed in lean, active young males with some IF protocols [13] warrant caution in recommending IF to this demographic without careful monitoring.

An important methodological caveat concerns the generalizability of findings from Ramadan fasting studies to secular IF protocols, such as TRE or ADF. Ramadan imposes a unique constellation of factors—nocturnal eating, circadian inversion, sleep curtailment, communal and spiritual dimensions—that are entirely absent from TRE or ADF. The reduction in sexual desire reported during Ramadan (Talib et al. [10]) therefore likely reflects circadian disruption, sleep deprivation, and psychosocial factors rather than metabolic fasting per se. Clinicians and researchers should therefore refrain from extrapolating Ramadan-derived sexual function outcomes to other IF regimens without explicit acknowledgment of these confounders.

### 4.2. Female Sexual Function: A Critical Knowledge Gap

The near-complete absence of validated sexual function assessments in women undergoing IF represents a major limitation of the current literature. While improvements in hyperandrogenism and insulin resistance in PCOS patients [8] would theoretically be expected to enhance sexual function—given the known associations between PCOS, reduced sexual satisfaction, and sexual dysfunction [36]—this hypothesis remains untested.

Future studies should incorporate validated instruments such as the Female Sexual Function Index (FSFI) to assess libido, arousal, lubrication, orgasm, satisfaction, and pain domains. Such assessments are particularly important in PCOS populations, where sexual dysfunction is prevalent and may improve with metabolic interventions.

The reasons for this evidence gap are multifactorial and warrant explicit discussion. First, most IF intervention studies have been designed with metabolic endpoints (body weight, insulin resistance, lipid profiles) as primary outcomes, with reproductive hormones as secondary endpoints; sexual function outcomes have not been incorporated into study designs. Second, assessment of female sexual function requires validated questionnaires (e.g., FSFI) and culturally sensitive research environments; cultural and social barriers may have discouraged inclusion of these outcomes in dietary intervention trials. Third, researchers may have implicitly assumed that hormonal improvements (e.g., reduced androgens in PCOS) would automatically translate to improved sexual function, without directly testing this hypothesis. Fourth, female sexual dysfunction has historically been understudied relative to male sexual dysfunction—a disparity that extends to dietary intervention research. Addressing this gap requires intentional inclusion of validated sexual function instruments as primary or co-primary endpoints in future IF trials, with particular attention to PCOS populations where sexual dysfunction is prevalent [36] and IF shows the strongest evidence for hormonal benefit [8,26,27].

The absence of validated female sexual function data under IF conditions represents a critical gap that limits the clinical applicability of this review. The Female Sexual Function Index (FSFI), a 19-item validated questionnaire assessing six domains (desire, arousal, lubrication, orgasm, satisfaction, and pain), has been widely used in dietary and metabolic intervention trials, yet no IF study has incorporated it as a primary or secondary endpoint. This omission is particularly consequential given that women with PCOS—the population most studied in IF research—have significantly higher rates of sexual dysfunction compared to age-matched controls [36]. The mechanisms by which IF improves insulin sensitivity and reduces hyperandrogenism in PCOS could plausibly translate into improvements in sexual function; however, this hypothesis remains entirely untested. Future IF trials in women should incorporate the FSFI or equivalent instruments as mandatory secondary endpoints.

### 4.3. PCOS: The Strongest Evidence for Reproductive Benefits

The most compelling evidence for IF benefits in reproductive health comes from studies in women with PCOS. The improvements in hyperandrogenism, insulin resistance, and menstrual regularity observed with TRF [8] address core pathophysiological features of PCOS and suggest potential fertility benefits.

The mechanisms underlying these benefits involve: (1) improved insulin sensitivity, reducing insulin-driven ovarian androgen production; (2) increased SHBG, reducing free androgen bioavailability; (3) weight loss and reduced adiposity, improving leptin signaling and reducing peripheral androgen production; and (4) potential circadian rhythm optimization, particularly with earlier eating windows [13].

However, several important questions remain: (1) What is the optimal IF protocol (timing, duration, degree of restriction) for PCOS? (2) How do IF effects compare to other dietary interventions (Mediterranean diet, low-glycemic diet) or pharmacological treatments (metformin, inositol)? (3) Do improvements in hormonal and metabolic parameters translate to enhanced fertility outcomes (pregnancy, live birth)? (4) Are benefits sustained long-term or does adaptation occur?

### 4.4. Mechanistic Insights and Sexually Dimorphic Responses

The reviewed evidence highlights multiple mechanistic pathways through which IF influences sexual and reproductive function, including HPG axis modulation, insulin–adipokine signaling, SHBG regulation, oxidative stress pathways, and neurotransmitter systems (Figure 2).

Importantly, these mechanisms appear to operate differently depending on baseline metabolic status. In metabolically compromised states (obesity, PCOS, HFD-induced dysfunction), IF corrects underlying pathophysiology and improves reproductive function. In metabolically healthy or energy-deficient states, IF may suppress the HPG axis as an adaptive energy conservation response, potentially impairing reproduction.

Sexually dimorphic responses are evident, with females appearing more vulnerable to adverse reproductive effects of IF under certain conditions [9,20,28]. This may reflect: (1) greater energetic costs of female reproduction (pregnancy, lactation); (2) more complex hormonal regulation of the female reproductive cycle; (3) tighter coupling between metabolic status and female fertility; and (4) potential sex differences in metabolic responses to fasting.

### 4.5. Animal Studies: Insights and Limitations

Animal studies provide valuable mechanistic insights and demonstrate proof-of-concept for IF effects on reproductive function. The protective effects of IF against HFD-induced reproductive dysfunction [16,17,19] suggest potential therapeutic applications in metabolic disease. The molecular pathways identified—particularly SIRT1/NRF2 antioxidant signaling and NLRP3 inflammasome suppression [16]—represent targets for future investigation in humans.

However, several factors limit translatability of animal findings to human sexual function: (1) differences in reproductive physiology between rodents and humans; (2) use of extreme IF protocols (complete 24 h fasts) that differ from typical human IF practices; (3) short study durations relative to lifespan; (4) lack of assessment of complex sexual behaviors analogous to human sexual function; and (5) controlled laboratory conditions that do not reflect real-world human IF implementation.

### 4.6. Clinical Implications and Recommendations

Based on the current evidence, the following clinical recommendations can be made:

For obese adults: Moderate IF protocols, such as 16:8 TRE, appear safe from a reproductive hormone perspective and may be recommended as part of weight management strategies [12]. Patients should be counseled about potential transient reductions in libido, particularly during initial adaptation.

For women with PCOS: IF, particularly TRF with earlier eating windows, shows promise for improving hyperandrogenism and menstrual regularity and may be considered as an adjunct to standard PCOS management [8,26,27]. Implementation should be individualized, with monitoring of menstrual patterns, metabolic parameters, and symptoms.

For lean, young, or athletic individuals: Caution is warranted, as IF may suppress reproductive hormones and function in energy-deficient states [9,13]. If IF is pursued in these populations, careful monitoring of menstrual regularity (females), libido, and hormonal status is advised.

For couples attempting conception: Given limited data on fertility outcomes, IF should be implemented cautiously in individuals actively trying to conceive. In women with PCOS-related infertility, IF may be beneficial as part of preconception optimization but should be combined with appropriate fertility monitoring.

General considerations: IF protocols should be individualized based on age, sex, baseline metabolic status, reproductive goals, and overall health. Adequate nutritional intake during eating windows is essential to prevent energy deficiency. Circadian alignment (earlier eating windows) may optimize metabolic and hormonal benefits [13].

### 4.7. Limitations of Current Evidence

Several limitations constrain interpretation and clinical application of current evidence:

1. Small sample sizes: Most human studies enrolled fewer than 100 participants, limiting statistical power and generalizability.
2. Short follow-up: Most interventions lasted weeks to months, providing no information on long-term effects or sustainability.
3. Heterogeneous protocols: Wide variation in IF protocols (timing, duration, degree of restriction) complicates comparison across studies and identification of optimal approaches.
4. Lack of validated sexual function measures: Most studies assessed hormones rather than actual sexual function, leaving the clinical significance of hormonal changes uncertain.
5. Absence of fertility endpoints: Direct measures of fertility (time to pregnancy, pregnancy rates, live birth rates) are lacking.
6. Predominance of animal data: Many mechanistic insights derive from animal studies, with uncertain human relevance.
7. Publication bias: Positive findings may be preferentially published, potentially overestimating benefits.
8. Confounding factors: Many studies did not adequately control for weight loss, dietary composition, exercise, sleep, or psychosocial factors that may independently affect sexual function.

### 4.8. Future Research Directions

To advance understanding of IF effects on sexual function and provide evidence-based clinical guidance, future research should:

1. Conduct adequately powered RCTs with validated sexual function instruments (IIEF for males, FSFI for females) as primary outcomes.
2. Include diverse populations across age, BMI, metabolic health status, and reproductive life stages.
3. Standardize IF protocols to enable comparison across studies while also comparing different protocols head-to-head.
4. Assess long-term outcomes, including sustained effects on sexual function, fertility rates, and pregnancy outcomes.
5. Investigate mechanisms through comprehensive hormonal profiling, metabolomics, and assessment of adipokines, inflammatory markers, and oxidative stress in humans.
6. Examine sex differences systematically, with adequate representation of both sexes and sex-stratified analyses.
7. Evaluate circadian timing effects by comparing early vs. late eating windows.
8. Include quality of life and relationship measures to capture broader impacts of IF on sexual health and well-being.
9. Study special populations, including PCOS, metabolic syndrome, erectile dysfunction, and infertility patients.
10. Conduct comparative effectiveness research evaluating IF against other dietary interventions and standard treatments.

Prioritize assessment of female sexual function using validated instruments: Future RCTs should include the Female Sexual Function Index (FSFI) as a primary or co-primary outcome measure. Priority populations include women with PCOS (where IF shows the strongest hormonal benefit and sexual dysfunction is prevalent [36]), postmenopausal women (where hormonal changes may affect sexual function), and women with obesity (where metabolic improvements may translate to sexual health benefits). Studies should include psychosocial measures (body image, mood, relationship quality) alongside biological endpoints. Mechanistic studies should correlate IF-induced changes in androgens, estrogens, and insulin sensitivity with FSFI domain scores to establish biological plausibility.

Priority research agenda for female sexual function and IF: Future studies should (1) recruit premenopausal and postmenopausal women in separate cohorts to account for hormonal heterogeneity; (2) administer the FSFI or FSDS-R at baseline and at ≥12-week follow-up; (3) include both PCOS and non-PCOS participants with stratified analyses; (4) standardize IF protocol type (TRE 16:8 vs. 5:2 vs. ADF), duration (minimum 12 weeks), and caloric composition; (5) simultaneously measure hormonal endpoints (LH, FSH, estradiol, testosterone, AMH, SHBG, insulin, HOMA-IR) and validated sexual function scores; and (6) control for psychological confounders (body image, depression, relationship quality) that independently modulate female sexual function. Head-to-head comparison between TRE and ADF protocols in women with PCOS would be particularly informative given the differential effects on circadian biology and eating behavior.

## 5. Conclusions

Intermittent fasting exerts complex, context-dependent effects on sexual and reproductive function in males and females. In males, limited human evidence suggests preserved erectile function but potentially reduced sexual desire during fasting periods, with neutral effects on testosterone in obese adults undergoing moderate TRE. Animal studies demonstrate protective effects against diet-induced reproductive dysfunction but potential harm under prolonged energy restriction. In females, IF shows promise for improving hyperandrogenism and menstrual regularity in PCOS through enhanced insulin sensitivity and reduced free androgen index, though direct sexual function data are absent. Mechanistic pathways involve modulation of the HPG axis, insulin–adipokine signaling, SHBG, oxidative stress, and neurotransmitter systems, with sexually dimorphic and metabolic status-dependent responses.

Current evidence supports cautious use of IF in metabolically compromised populations, particularly women with PCOS, while warranting caution in lean, young, or energy-deficient individuals. Importantly, despite the title’s reference to ‘sexual function,’ it must be explicitly acknowledged that direct evidence specifically addressing female sexual function domains—including desire, arousal, lubrication, orgasm, satisfaction, and dyspareunia—is entirely absent from the current IF literature. All conclusions regarding female sexual function are therefore inferential, based on hormonal and metabolic surrogate endpoints. Furthermore, the evidence for reduced sexual desire in males derives almost exclusively from Ramadan fasting studies, which involve unique confounders (mandatory sexual abstinence, fluid restriction, nocturnal sleep disruption, religious context) that preclude generalization to other IF protocols, such as TRE and ADF. These represent critical evidence gaps that must be addressed in future research. Future research employing standardized IF protocols, validated sexual function instruments (FSFI for women, IIEF for men), diverse populations, and long-term fertility endpoints is essential to establish evidence-based clinical recommendations. Until such evidence is available, IF implementation for sexual health should be individualized, with careful monitoring of reproductive parameters and patient-reported outcomes.

Two critical limitations must be explicitly acknowledged: first, no published study has directly assessed female sexual function under IF conditions using validated instruments such as the FSFI, rendering conclusions about IF effects on female sexual function premature and speculative; second, findings from Ramadan fasting studies cannot be generalized to secular TRE or ADF protocols due to the unique cultural, spiritual, and chronobiological characteristics of Ramadan. These limitations are reflected in the manuscript title, which explicitly highlights the evidence gap in women. Dedicated prospective clinical trials incorporating validated sexual function instruments are urgently needed to address these gaps.

## Figures and Tables

**Table 1:** Comparison of intermittent fasting protocols and their effects on sexual function, reproductive hormones, and fertility outcomes in males and females.

IF Protocol	Fasting Pattern	Male Hormones	Female Hormones	Male Sexual Function	Female Sexual Function	Fertility Outcomes	Evidence Quality
TRE (16:8)	Daily 16 h fast, 8 h eating window	Testosterone: neutral in obese [12]; reduced in lean active males [13]	Androgens ↓, SHBG ↑ in PCOS [8,13]; neutral in obese/postmenopausal [12,24]	No data on libido/erectile function from TRE-specific studies; hormonal neutrality in obese [12]	No validated FSFI data; PCOS: improved hyperandrogenism, menstrual regularity [8]	PCOS: menstrual normalization >70% [8]; improved ovulatory markers [26,27]; no human pregnancy/live-birth data	MODERATE (1 RCT + 1 SR)
ADF	Alternate-day complete or modified fast (~25% kcal on fast days)	Protective vs HFD-induced dysfunction in animals [16,17]; no human hormone data	No human data identified	No human data	No data	Animal: protective vs. HFD-induced reproductive dysfunction [16,17]; no human data	LIMITED (animal data only)
5:2 Diet	5 normal days + 2 days ~500 kcal/week	No specific human hormone data identified	Mentioned in PCOS context [35]; no detailed results available	No data	No data	No direct fertility endpoint data	INSUFFICIENT
Ramadan Fasting	Daytime abstinence (food, fluid, sexual activity) ~12–14 h for 1 month; religious context	Testosterone: unchanged [10,14]; FSH ↓ [10]; transient fluctuations normalizing post-Ramadan [15]	LH, FSH, estradiol, testosterone, prolactin: unchanged in healthy women [23]	Sexual desire ↓ (*p* = 0.002) [10]; intercourse frequency ↓ [10]; erectile function preserved [10]. NOTE: sexual abstinence is religiously mandated; findings NOT generalizable to TRE/ADF	No validated FSFI data	No fertility endpoint data; reduced coital frequency may indirectly affect fertility [10]	LIMITED (1 RCT + 1 SR; Ramadan-specific confounders limit generalizability to other IF protocols)
IF-DR (animal)	Every-other-day food deprivation (12 weeks); rodent models	Testosterone ↓ (non-sig), LH/FSH ↓ [18]; spermatogenesis impaired [18]; protective under HFD [16,17]	Estradiol ↑, testosterone ↓, LH ↓; HPG axis suppression; disrupted estrous cyclicity [9]; greater female vulnerability [20]	Sperm count ↓, testicular weight ↓ under energy restriction [18]	Disrupted estrous cycle, ovarian changes [9]; greater female vulnerability than males [20]	Impaired spermatogenesis [18]; disrupted ovulatory cyclicity [9]; protective under HFD [16,17]	LIMITED (animal data; extreme protocols; uncertain human translatability)

Abbreviations: TRE = time-restricted eating; ADF = alternate-day fasting; IF-DR = intermittent fasting dietary restriction; HFD = high-fat diet; PCOS = polycystic ovary syndrome; SHBG = sex hormone-binding globulin; FSFI = Female Sexual Function Index; SR = systematic review; RCT = randomized controlled trial; HPG = hypothalamic–pituitary–gonadal axis. Numbers in brackets correspond to reference citations in the main text. Evidence quality definitions: STRONG = multiple well-designed human RCTs; MODERATE = human observational studies or single RCTs with limitations; LIMITED = small studies, animal data, or inconsistent findings; INSUFFICIENT = inadequate data.

## Data Availability

No new data were created or analyzed in this study. Data sharing is not applicable to this article.

## References

[1] Prosowski M., Kołodziejczyk J., Szymański M., Szymański M., Szymański M. (2025). Intermittent fasting—Its benefits and risks. Arch. EuroMedica.

[2] Shkorfu W., Fadel A., Hamsho M., Ranneh Y., Shahbaz H.M. (2025). Intermittent Fasting and Hormonal Regulation: Pathways to Improved Metabolic Health. Food Sci. Nutr..

[3] Rius-Bonet J., Macip S., Closa D., Massip-Salcedo M. (2024). Intermittent fasting as a dietary intervention with potential sexually dimorphic health benefits. Nutr. Rev..

[4] Rosen R.C., Bachmann G.A. (2008). Sexual well-being, happiness, and satisfaction, in women: The case for a new conceptual paradigm. J. Sex Marital Ther..

[5] Esposito K., Giugliano F., Di Palo C., Giugliano G., Marfella R., D’Andrea F., D’Armiento M., Giugliano D. (2004). Effect of lifestyle changes on erectile dysfunction in obese men: A randomized controlled trial. JAMA.

[6] Plant T.M., Zeleznik A.J. (2015). Knobil and Neill’s Physiology of Reproduction.

[7] Schneider J.E., Brozek J.M., Keen-Rhinehart E. (2014). Our stolen figures: The interface of sexual differentiation, endocrine disruptors, maternal programming, and energy balance. Horm. Behav..

[8] Güven A. (2023). Eight-Hour Time-Restricted Feeding: A Strong Candidate Diet Protocol for First-Line Therapy in Polycystic Ovary Syndrome. Nutrients.

[9] Kumar S., Kaur G. (2013). Intermittent fasting dietary restriction regimen negatively influences reproduction in young rats: A study of hypothalamo-hypophysial-gonadal axis. PLoS ONE.

[10] Talib R.A., Khalafalla K., Canguven O. (2015). The effect of fasting on erectile function and sexual desire on men in the month of Ramadan. Urol. J..

[11] Huynh L.M., Balasubramanian A., Ostrowski K.A., Helo S., Ramasamy R., Lipshultz L.I. (2020). Organic diet and intermittent fasting are associated with improved erectile function. Urology.

[12] Cienfuegos S., Corapi S., Gabel K., Ezpeleta M., Kalam F., Lin S., Pavlou V., Varady K.A. (2024). Effect of time restricted eating versus daily calorie restriction on sex hormones in males and females with obesity. medRxiv.

[13] Cienfuegos S., Gabel K., Kalam F., Ezpeleta M., Varady K.A. (2022). Effect of Intermittent Fasting on Reproductive Hormone Levels in Females and Males: A Review of Human Trials. Nutrients.

[14] Al-Chalabi H.K. (2013). Effect of Ramadan Fasting on Sex Hormones in Infertile Male. Tikrit J. Pharm. Sci..

[15] Poursalehian M., Soltanieh S., Barzegar A., Hassanzadeh Keshteli A., Esmaillzadeh A., Adibi P. (2024). Impact of Ramadan fasting on serum levels of major endocrinology hormonal and biochemical parameters in healthy non-athlete adults: A systematic review and meta-analyses. PLoS ONE.

[16] Hemead H.M., Hamed M.F., Saleh A.A., El-Sayed Y.S. (2025). Intermittent fasting restores fertility dysfunction caused by a high-fat diet in male rats: Role of SIRT-1/NRF2/P38 MAPK/NLRP3. Reprod. Fertil. Dev..

[17] Buranaamnuay K., Changsangfa C., Ruschadaariyachat S. (2025). Intermittent fasting is beneficial for body weight regulation and reproductive phenotypes in high-fat diet-fed male mice. Discov. Med..

[18] Omolaso B.O., Ajayi I.E., Oyeyemi W.A., Farombi T.H., Ajayi O.L., Soladoye A.O. (2012). Evaluation of the Effects of Fasting on Fertility in Adult Male Wistar Rats. IOSR J. Pharm. Biol. Sci..

[19] Üstündağ Ü.V., Özer A., Koca H.B., Koca O., Karaman M.İ., Güneş M. (2023). Exploring the Impact of Ketogenic Diet and Intermittent Fasting on Male Rats’ Testicular Health: An Analysis of Hormonal Regulation, Oxidative Stress, and Spermatogenesis. J. Food Biochem..

[20] Oyelowo O.T., Adekunbi D.A., Adedeji A.L. (2022). Skipping the first active meal appears to adversely alter reproductive function in female than male rats. Curr. Res. Physiol..

[21] Xie K., Wang C., Scifo E., Pearson B., Ryan D., Henzel K., Markert A., Schaaf K., Mi X., Tian X. (2025). Intermittent fasting boosts sexual behavior by limiting the central availability of tryptophan and serotonin. Cell Metab..

[22] Sayme N., Patel P., Ramasamy R., Kavoussi P. (2023). The impact of intermittent fasting on sperm parameters. Fertil. Steril..

[23] Caglayan E.K., Engin-Ustun Y., Sari N., Karacavus S., Seckin L. (2014). Effects of Long-Term Fasting on Female Hormone Levels: Ramadan Model. Clin. Exp. Obstet. Gynecol..

[24] Kalam F., Gabel K., Cienfuegos S., Wiseman E., Ezpeleta M., Pavlou V., Lin S., Varady K.A. (2022). Effect of time-restricted eating on sex hormone levels in premenopausal and postmenopausal females. Obesity.

[25] Azziz R., Carmina E., Chen Z., Dunaif A., Laven J.S., Legro R.S., Lizneva D., Natterson-Horowtiz B., Teede H.J., Yildiz B.O. (2016). Polycystic ovary syndrome. Nat. Rev. Dis. Primers.

[26] Velissariou I., Davoulou P., Evangelou E., Karampela I., Dalamaga M., Pergialiotis V. (2025). The impact of intermittent fasting on fertility: A focus on polycystic ovary syndrome and reproductive outcomes in Women—A systematic review. Metab. Open.

[27] Ranneh Y., Akim A.M., Hamid H.A., Khazaai H., Fadel A., Zakaria Z.A., Albujja M., Bakar M.F.A. (2025). Effect of Intermittent Fasting on Anthropometric Measurements, Metabolic Profile, and Hormones in Women with Polycystic Ovary Syndrome: A Systematic Review and Meta-Analysis. Nutrients.

[28] Waly T.A., Nugroho B.S. (2025). The analysis study effect of intermittent fasting on female reproductive hormones and menstrual cycle: A comprehensive systematic review. Int. J. Med. Sci. Health Res..

[29] Yu Y., Deng C., Huang X., Guo X., Li Y. (2021). Intermittent fasting ameliorates di-(2-ethylhexyl) phthalate-induced precocious puberty in female rats: A study of the hypothalamic–pituitary–gonadal axis. Reprod. Biol..

[30] Ehteram H., Ramezani M., Moghimian M., Salmani H., Bibak B., Rashidi I. (2017). Effect of Intermittent Feeding on Gonadal Function in Male and Female NMRI Mice During Chronic Stress. Braz. Arch. Biol. Technol..

[31] Diamanti-Kandarakis E., Dunaif A. (2012). Insulin resistance and the polycystic ovary syndrome revisited: An update on mechanisms and implications. Endocr. Rev..

[32] Tena-Sempere M. (2008). Ghrelin as a pleotrophic modulator of gonadal function and reproduction. Nat. Clin. Pract. Endocrinol. Metab..

[33] Agarwal A., Aponte-Mellado A., Premkumar B.J., Shaman A., Gupta S. (2012). The effects of oxidative stress on female reproduction: A review. Reprod. Biol. Endocrinol..

[34] Yang X., Liu W., Zhuo Y., Luo T., Wu D., Hua L. (2025). Intermittent Fasting in Female Reproduction: A Double-Edged Sword. Nutr. Rev..

[35] Salamy N., Putri A.K., Sari D.P. (2025). Pengaruh puasa intermiten terhadap kesehatan hormonal dan reproduksi wanita: Tinjauan literatur. J. Penelit. Keperawatan Kontemporer.

[36] Stovall D.W., Scriver J.L., Clayton A.H., Williams C.D., Pastore L.M. (2012). Sexual function in women with polycystic ovary syndrome. J. Sex. Med..

